# Lung adenocarcinoma and anti‐transcriptional intermediary factor 1‐gamma positive dermatomyositis complicated with spontaneous oesophageal rupture

**DOI:** 10.1002/rcr2.403

**Published:** 2019-02-04

**Authors:** Takeshi Saraya, Masaki Tamura, Keisuke Kasuga, Masachika Fujiwara, Hajime Takizawa

**Affiliations:** ^1^ Department of Respiratory Medicine Kyorin University School of Medicine Tokyo Japan

**Keywords:** Anti‐transcriptional intermediary factor 1‐γ antibody, dermatomyositis, dysphagia, lung cancer, spontaneous oesophageal rupture

## Abstract

A 58‐year‐old man presented with a two‐month history of facial erythema and dry cough. Physical examination revealed typical cutaneous manifestations of dermatomyositis (DM), including heliotrope rash and shawl sign. A chest X‐ray revealed a 4‐cm mass in the right middle lung. After bronchoscopy and investigation of auto‐antibodies, he was diagnosed with co‐occurring transcriptional intermediary factor 1‐gamma (TIF1‐γ) positive DM and lung adenocarcinoma. He was administered oral prednisolone for subsequent muscle weakness, but developed TIF1‐γ positive DM‐associated oropharyngeal dysphagia complicated by spontaneous oesophageal rupture and died from progression of chemoresistant lung cancer.

## Introduction

Inflammatory myopathies have several myositis‐associated and myositis‐specific auto‐antibodies 1. Among them, anti‐Mi‐2, anti‐melanoma differentiation‐associated gene 5 (MDA5), and anti‐transcriptional intermediary factor 1‐γ (TIF1‐γ) antibodies have only recently become commercially available in Japan since December 2016. Hence, clinical diagnosis of the symptoms associated with these auto‐antibodies might still be underestimated by general physicians. Herein, we report a unique case of co‐occurring TIF1‐γ positive dermatomyositis (DM) and lung adenocarcinoma, who also suffered from muscle weakness in extremities, and elevated serum muscle enzyme levels, oropharyngeal dysphagia followed by spontaneous oesophageal rupture.

## Case Report

**Figure 1:**
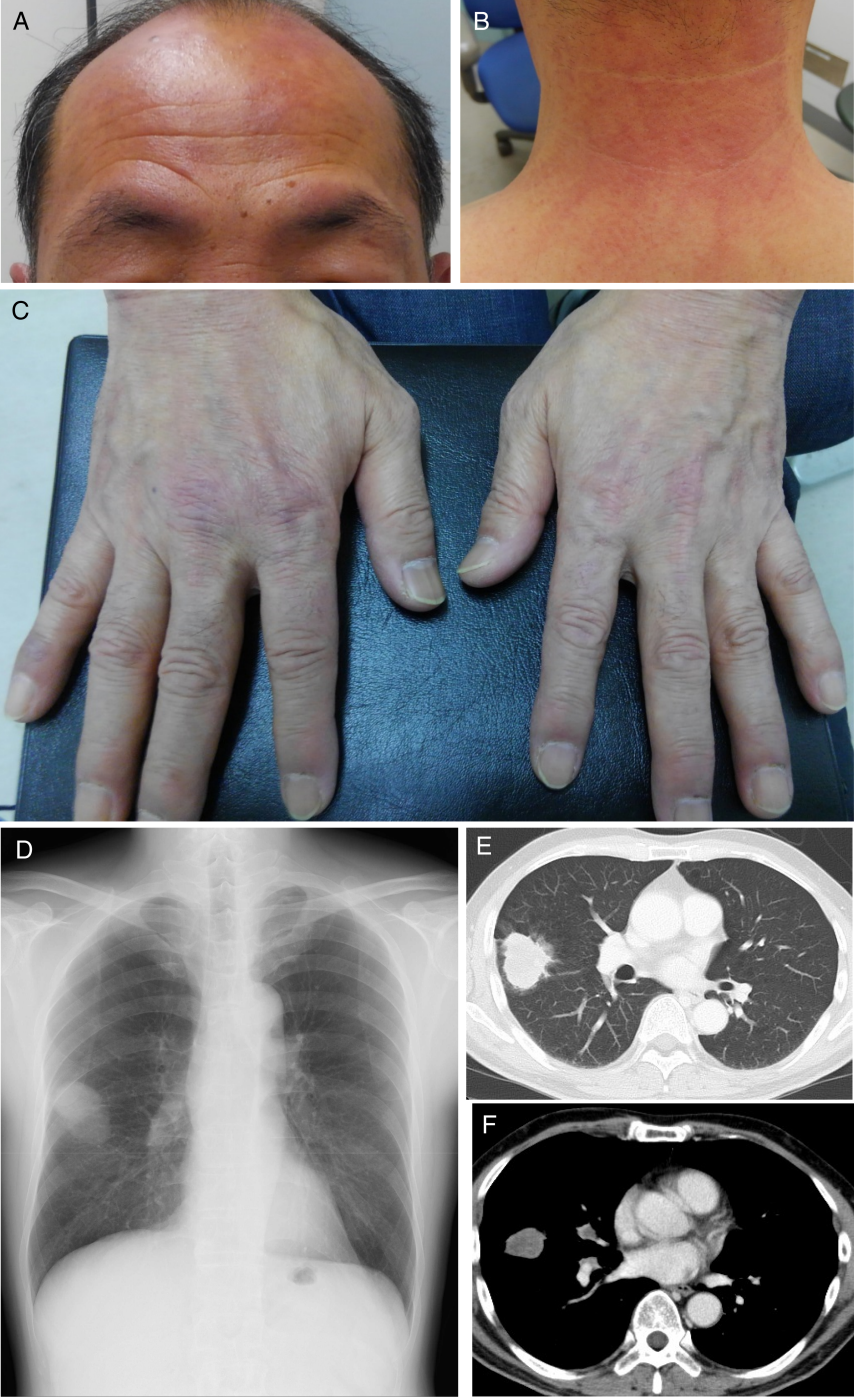
Macular rash on his forehead and swelling of upper eyelids (A), suggestive of a heliotrope rash. Shawl sign was recognized characterized by a widespread, flat, reddened area on his upper back, shoulders, and posterior neck (B). A flat, red rash on the back of the fingers and hands, indicating a Gottron’s sign (C). Chest X‐ray demonstrated the mass as large as 4 cm in diameter in the right middle lung fields (D), which was confirmed by thoracic computed tomography depicted as an inhomogeneously enhanced solitary mass (4 cm in size) in the right upper lobe (E), with ipsilateral hilar lymphadenopathy (F).

A 58‐year‐old man was admitted to our hospital with a two‐month history of facial erythema and dry cough. He had no remarkable medical history except for essential hypertension five years ago. He was a current smoker with a smoking history of 37 pack‐years. Although he initially seemed well and his vital signs were normal, a thorough physical examination revealed characteristic cutaneous manifestations of DM. He had a macular rash along with swelling on his forehead and upper eyelids (Fig. 1A), suggestive of a heliotrope rash. He also displayed a shawl sign characterized by a widespread, flat, reddened area on his upper back, shoulders, and posterior neck (Fig.1B). Additionally, he had a flat, red rash on the back of the fingers and hands, indicating a Gottron’s sign (Fig. 1C). We observed a marked elevation of muscle enzymes in his serum, including aspartate transaminase (294 IU/L), creatine kinase (7833 IU/L), aldolase (50.3 U/L), and lactase dehydrogenase (606 IU/L) (Table 1). Soon after admission, he felt muscle weakness, but not muscle pain, in his extremities. Manual muscle test detected reduced strength in his bilateral deltoid and hamstring muscles (grade 4/4), as well as iliopsoas muscles (grade 2/3), suggesting proximal muscle impairment. Based on the suspicion of idiopathic inflammatory myopathies, further analysis of auto‐antibodies in patient’s serum revealed the presence of TIF1‐γ auto‐antibodies, but not that of anti‐aminoacyl‐tRNA synthetases (ARS), including anti‐Jo‐1, anti‐PL7, anti‐PL12, anti‐EJ, anti‐OJ, anti‐KS, melanoma differentiation‐associated gene 5, and anti‐Mi‐2 (Table 1). Thus, the patient was tentatively diagnosed with possible DM and TIF1‐γ positive myopathy. Fat suppressed T2‐weighted magnetic resonance imaging coronal image demonstrated a high‐intensity lesion in the bilateral rectus femoris, right vastus lateralis, vastus medialis, and bilateral obturator muscle. Needle electroneuromyography showed a myopathic pattern, with motor unit potentials diminished in amplitude as well as duration. Based on the DM diagnostic criteria recommended by Bohan & Peter and The Research Committee of the Japanese Ministry of Health and Welfare in 2015, we diagnosed the patient with TIF1‐γ positive DM.

**Table 1:** Serum laboratory examination on admission.

WBC	9200 (/μL)	PT‐INR	0.91
RBC	513 (×10^4^/μL)	APTT	37.4 (sec)
Hb	16.8 (g/dL)	Fib	676 (mg/dL)
Ht	50.3 (%)	D‐dimmer	0.59 (μg/mL)
Plt	16.3 (×10^4^/μL)	ANA	40×
TP	5.9 (g/dL)	Anti‐ARS	Negative
Alb	3.3 (g/dL)	RF	Negative
AST	294 (IU/L)	Anti‐MDA5	<5
ALT	137 (IU/L)	Anti‐Mi‐2	<5
LDH	606 (IU/L)	Anti‐TIF1‐γ	123
CK	7833 (IU/L)	Anti‐Jo1	Negative
Cr	0.78 (mg/dL)	Anti‐Ro	Negative
ALD	50.3 (U/L)	Anti‐La	Negative
CRP	1.91 (mg/dL)		

Alb, albumin; ALD, aldolase; ALT, alanine aminotransferase; ANA, anti‐nuclear antibody; APTT, activated partial thromboplastin time; ARS, aminoacyl‐tRNA synthetases; AST, aspartate transaminase; CK: creatine kinase; Cr, creatinine; CRP, C‐reactive protein; Hb, haemoglobin; Ht, haematocrit; INR, international normalized ratio; LDH, lactate dehydrogenase; MDA‐5, melanoma differentiation‐associated gene 5; Plt, platelet; PT, prothrombin time; RBC, red blood cell; RF, rheumatoid factor; TIF1‐γ, transcriptional intermediary factor 1‐gamma; TP, total protein; WBC, white blood cell.

In addition, a routine chest X‐ray (Fig. 1D) performed during admission showed a mass in the right middle lung field. Thoracic and abdominal contrast‐enhanced computed tomography (CT) identified an inhomogeneously enhanced solitary mass (4 cm in size) in the right upper lobe (Fig. 1E), with ipsilateral hilar lymphadenopathy (Fig. 1F) as well as liver and left adrenal metastasis. Subsequent bronchoscopy and tumour biopsy confirmed lung adenocarcinoma. Thereby, the patient was diagnosed with cT2bN1M1b (stage IV) lung adenocarcinoma combined with TIF1‐γ positive DM.

Following diagnosis, he was treated with 75 mg/day oral prednisolone for myopathy, which alleviated his muscle weakness, and improved his serum muscle enzymes and skin lesions within two weeks. At day 12 from admission, the patient was administered intravenous chemotherapy with cisplatin, pemetrexed sodium hydrate, and bevacizumab for lung adenocarcinoma. However, on day 19, he developed dysphagia, which was confirmed by a videofluoroscopic swallow study. He displayed hypopharyngeal muscle weakness, dysfunction of laryngeal closure, and ineffective oesophageal motility. Over the following one month, his posterior wall of the oesophagus at the level of the entrance considered to be ruptured due to emergence of oesophagus diverticulum on a repeated videofluoroscopic swallow study along with advent of both cervical subcutaneous emphysema on chest X‐ray or the air in the cervical oesophageal wall on cervical CT. Thereafter, his general condition gradually deteriorated. Although the weakness in his extremities and his skin lesions were controlled, his oropharyngeal dysphagia persisted with conservative therapy, and his lung tumour was resistant to chemotherapy, resulting in his death due to respiratory failure six months later.

## Discussion

Approximately 70% of DM patients are positive for auto‐antibodies such as anti‐ARS (20%), anti‐MDA (20%), anti‐Mi‐2 (10%), and anti‐TIF1‐γ (20%). However, the latter three auto‐antibodies have only recently became commercially available in Japan (since December 2016), resulting in under‐diagnoses of clinical symptoms related to these auto‐antibodies by the Japanese general physicians. In addition, while patients with anti‐TIF1‐γ positive DM were less likely to have systemic manifestations such as interstitial lung disease, Raynaud phenomenon, and arthritis/arthralgia, they have extensive skin disease and high risk for malignancy (up to 75%), especially in patients aged >40 years, as observed in our patient. Another report described that patients with anti‐TIF1‐γ positive DM have 27‐fold higher odds of having cancer than those who were negative for anti‐TIF1‐γ 2. In this regard, our patient presented with co‐occurring TIF1‐γ auto‐antibody positive DM and lung adenocarcinoma, resulted in spontaneous oesophageal rupture was characterized as following points.

Firstly, marked elevation of muscle enzyme levels can be seen in DM patients with or without cancer. However, the highly elevated muscle enzyme levels of our case subsided and alleviated his muscle weakness in extremities soon after initiation of steroid therapy. This was considered to be as a result of the anti‐TIF1‐γ DM and not cancer‐associated myopathy (CAM) (i.e. paraneoplastic syndrome) or tumour lysis syndrome. In the clinical context of elevated muscle enzyme levels with muscle weakness, the positive myositis‐associated antibodies, such as anti‐HMGCR, anti‐TIF1‐γ, anti‐SAE, anti‐NXP2, anti‐Mi‐2, raises concerns about identification of cancer 3.

Secondly, persistent dysphagia could be potentially associated as an atypical phenomenon of anti‐TIF1‐γ DM. Mugii et al. 4 reported that 84.6% of DM patients with dysphagia were positive for anti‐TIF1‐γ auto‐antibody when compared to those with no dysphagia (19.0%), suggesting that anti‐TIF1‐γ auto‐antibody can be an independent risk factor (odds ratio 11.8) for dysphagia, as observed in our patient. In this point of view, we cannot exclude the aspect of CAM, because the DM patients with CAM tend to have dysphagia (44%) rather than those of non‐cancer (14.5%) 5.

Thirdly, there has been only one case report describing the spontaneous oesophageal rupture in adult DM 6 who was successfully treated with total oesophagectomy. In general, spontaneous oesophageal rupture, named Boerhaave syndrome, is a spontaneous perforation of the oesophagus that results from a sudden increase in intra‐oesophageal pressure combined with negative intrathoracic pressure (i.e. severe straining or vomiting). A subset of patient with Boerhaave syndrome has underlying eosinophilic oesophagitis, Barrett’s or infectious ulcers, and medication‐induced oesophagitis. This raises the concerns about the possibility of bevacizumab‐induced oesophagitis in the present case. However, to the best of our knowledge, only one case of bevacizumab‐induced oesophagitis has been reported until today 7. The reported case showed aorto‐oesophageal fistula rupture during the chemotherapy include bevacizumab following colon cancer resection, but never had dysphagia or laryngopharyngeal muscle weakness, and dysfunction of the oesophageal motility 7. Furthermore, Boerhaave syndrome usually involves the left posterolateral aspect of the distal intrathoracic oesophagus and extends for several centimetres, while the present case had perforation at the entrance of the oesophagus, showing dysfunction of the inferior pharyngeal muscle or upper oesophageal dysmotility possibly due to DM. Thus, we present a rare case of anti‐TIF1‐γab‐positive DM co‐occurring with lung adenocarcinoma, along with refractory dysphagia followed by spontaneous oesophageal rupture.

In conclusion, this case exemplifies that physicians should consider the possibility of TIF1‐γ auto‐antibody‐associated DM occurring in patients with malignancies and/or oropharyngeal dysphagia including spontaneous oesophageal rupture.

### Disclosure Statement

Appropriate written informed consent was obtained for publication of this case report and accompanying images.
